# Multiplying the efficiency and impact of biofortification through metabolic engineering

**DOI:** 10.1038/s41467-020-19020-4

**Published:** 2020-10-15

**Authors:** Dominique Van Der Straeten, Navreet K. Bhullar, Hans De Steur, Wilhelm Gruissem, Donald MacKenzie, Wolfgang Pfeiffer, Matin Qaim, Inez Slamet-Loedin, Simon Strobbe, Joe Tohme, Kurniawan Rudi Trijatmiko, Hervé Vanderschuren, Marc Van Montagu, Chunyi Zhang, Howarth Bouis

**Affiliations:** 1 Laboratory of Functional Plant Biology, Department of Biology, Ghent University, K.L. Ledeganckstraat 35, B-9000 Ghent, Belgium; 2 Department of Biology, Institute of Molecular Plant Biology, ETH Zurich, Universitaetstrasse 2, 8092 Zurich, Switzerland; 3 Department of Agricultural Economics, Ghent University, Coupure Links 653, B-9000 Ghent, Belgium; 4 Advanced Plant Biotechnology Center, National Chung Hsing University, Taichung, Taiwan; 5 Donald Danforth Plant Science Center, St. Louis, MO 63132 USA; 6 HarvestPlus c/o IFPRI, Washington, DC USA; 7 Department of Agricultural Economics and Rural Development, University of Goettingen, Platz der Goettinger Sieben 5, 37073 Goettingen, Germany; 8 International Rice Research Institute, Manila, The Philippines; 9 International Center for Tropical Agriculture, CIAT, Cali, Colombia; 10 Tropical Crop Improvement Lab, Department of Biosystems, KU Leuven, Heverlee, Belgium; 11 Plant Genetics, TERRA Teaching and Research Center, Gembloux Agro-Biotech, University of Liège, Gembloux, Belgium; 12 International Plant Biotechnology Outreach, B-9052 Zwijnaarde, Belgium; 13 Biotechnology Research Institute, Chinese Academy of Agricultural Sciences, Beijing, China; 14 International Food Policy Research Institute, Washington, DC USA

**Keywords:** Metabolic engineering, Agricultural genetics, Molecular engineering in plants, Agriculture

## Abstract

Ending all forms of hunger by 2030, as set forward in the UN-Sustainable Development Goal 2 (UN-SDG2), is a daunting but essential task, given the limited timeline ahead and the negative global health and socio-economic impact of hunger. Malnutrition or hidden hunger due to micronutrient deficiencies affects about one third of the world population and severely jeopardizes economic development. Staple crop biofortification through gene stacking, using a rational combination of conventional breeding and metabolic engineering strategies, should enable a leap forward within the coming decade. A number of specific actions and policy interventions are proposed to reach this goal.

## Abstract (web summary)

Biofortification is an effective means to reduce micronutrient malnutrition. Here, the authors review recent advances in biofortification and propose stacking multiple micronutrient traits into high-yielding varieties through the combination of conventional breeding and genetic engineering approaches.

## Introduction

Micronutrient malnutrition or hidden hunger, the insufficient intake of vitamins and minerals, has a detrimental impact on human health. Hidden hunger affects more than two billion people worldwide, and has the highest prevalence in the African continent and South Asia. Children and women of reproductive age are most vulnerable to malnutrition, leading to stunted growth, health problems, and several birth related issues. Though seven minerals (iron, zinc, copper, calcium, magnesium, selenium, and iodine^1^) and several vitamins^2^ are often lacking in human diets, iron, zinc, vitamin A, and vitamin B9 (folate) deficiencies are amongst the most severe^3–5^. Biofortification, the elevation of the levels of micronutrients in food crops through agricultural technologies, is advocated as a pivotal means to reduce micronutrient malnutrition^6^.

Impressive progress has been made in biofortification of mainly single micronutrients across an array of primary staple food crops^6,7^. Biofortified crops have been developed via conventional breeding or genetic engineering, although the latter have yet to receive full approval for release to farmers. Biofortification via genetic engineering can enable high level accumulation of micronutrients and is not constrained by variation in available germplasm. Biofortification strategies can combine conventional breeding with genetic engineering. Genetic engineering enables simultaneous augmentation of multiple micronutrients, along with improving the post-harvest stability of vitamins, whilst also including agronomically important traits, such as enhanced yield and stress resilience.

In the last few years, the cost-effectiveness and feasibility of implementing biofortification using conventional breeding techniques has been established as a key intervention to reduce mineral and vitamin deficiencies in developing countries^8^. Consequently, a recent World Bank report takes the position that biofortified cereal crops should be the norm rather than the exception^9^.

HarvestPlus, which is a program under the Consultative Group on International Agricultural Research (CGIAR) Research Program on Agriculture for Nutrition and Health, is an umbrella of institutions working on biofortification in low- and middle-income countries. HarvestPlus collaborating institutions have developed, tested, released, and promoted the uptake by farmers and consumers of the following crops: zinc rice, zinc wheat, zinc maize, provitamin A maize, provitamin A cassava, iron beans, and iron pearl millet^7^. Approximately 8.5 million farm households across Africa, Asia, and Latin America are growing these biofortified crops (https://www.harvestplus.org). In addition, under the sponsorship of the International Potato Center (CIP), CIP and its partners have led the development, testing, and promotion of orange (high provitamin A) sweet potato^10^. CIP estimates that nearly seven million farm households, primarily in Africa, are now growing and consuming orange sweet potato (https://www.cipotato.org).

Through their combined efforts, biofortified crops are now released in 40 countries globally.

Additionally, a few other institutions have successfully worked on particular aspects of the development and testing of specific crops to achieve biofortification with minerals and vitamins through conventional breeding. However, these individual efforts are awaiting incorporation into breeding programs or to field applications (for a review, see Garg et al.^6^).

Despite wide acceptance of the effectiveness of biofortification and demonstrated high return on investment, the increase of only one nutrient per crop (either iron or zinc or provitamin A) remains a limiting factor– for example high zinc wheat, or high provitamin A maize. The breeding and release process for the first wave of biofortified varieties took 8–10 years. In a small number of cases, there is sufficient germplasm variation within the target crop species to add a second nutrient (e.g. high zinc into high provitamin A maize) using conventional breeding techniques, but this is expected to take an additional 8–10 years.

Metabolic engineering through transgenic technology enables introducing multiple biofortification traits, including high iron, high zinc, high provitamin A, or high folate, to most biofortified crop varieties relatively quickly – that is, in significantly <8–10 years. It may also be possible to reduce or stop the decrease of vitamin densities after harvest and during storage, which occurs due to exposure to light, oxygen, and/or high temperature. Furthermore, multi-biofortification can not only create crop products with stable enhanced micronutrients but also take advantage of the most profitable, highest yielding, and locally best-adapted varieties.

## Hidden hunger is a huge problem for human health and economies

Hidden hunger is the direct result of insufficient acquisition of minerals and vitamins from the diet. An estimated 2 billion people in the developing world suffer from health effects caused by hidden hunger (mainly related to deficiencies of iron, zinc, vitamin A, and folates (vitamin B9))^11^. This is primarily the result of poor-quality diets consisting of adequate energy consumption from inexpensive staple crops, but little consumption of expensive vegetable, fruit, pulses, and animal products that are richer in bioavailable minerals and vitamins. Inadequate intakes of essential vitamins and minerals affect disease resistance, cognitive development, physical growth, work productivity, and survival rate. Preschool children and women in their reproductive age are most vulnerable to deficiencies because they require higher micronutrient intakes^12^. Agricultural systems in developing countries are not providing sufficient minerals and vitamins at affordable prices for optimal nutrition and health. Supplements often do not reach the populations in need^13^. As impaired micronutrient status aggravates susceptibility to infectious diseases including COVID-19^14,15^, the current pandemic further underscores the need to improve the nutritional status of poor rural populations and increase their nutritional self-sufficiency through staple crop biofortification.

To combat vitamin A deficiency, for example, 10 billion vitamin A capsules (at an approximate cost of US dollar 10–15 billion) have been distributed over the past twenty years to preschool children – with the effect of reducing preschool mortality by an estimated 12–24%^16^. In addition, the Global Burden of Disease Study 2015 estimates that 1.5 billion people suffer from iron (Fe) deficiency anemia, which impairs cognitive function in preschool children^17^. Nearly 1.2 billion people are also at risk of zinc deficiency, which is associated with weakened immune systems and higher mortality^18^. Stunting, which likely results from zinc (Zn) deficiency as well, affects one out of four children under the age of five. Stunting is strongly associated with poor brain development and cognitive function^19^. Moreover, folate deficiency, which causes neural tube defects (NTDs) and megaloblastic anemia, and also aggravates iron deficiency anemia, is a highly underestimated form of hidden hunger. Worldwide, at least 300,000 births are affected by NTDs annually, the majority of which are caused by inadequate maternal folate status^20^. This number is largely underestimated because of a paucity of data on stillbirths and elective terminations, as well as lacking surveillance systems in low- and middle-income countries.

Progress in achieving the global targets of reducing child stunting by 40% and anemia by 50% by 2025 is still far too slow^21^. Global losses in economic productivity due to macronutrient and micronutrient deficiencies reach more than 3% of GDP^22^ at a global cost of US dollar 1.4-2.1 trillion per year^3,23^. Micronutrient malnutrition poses a great threat to global human health and economic development. Therefore, eliminating or at least restricting its occurrence is of utmost importance, which is in line with the United Nations Sustainable Developmental Goal 2 (UN-SDG2) i.e. achieving zero hunger.

## Crop biofortification using conventional breeding techniques

Biofortification aims at increasing the content of one or multiple micronutrients in staple food crops through agricultural technologies. In some cases, conventional breeding can be utilized to achieve this goal, thereby ensuring higher vitamin or mineral intake of the poor populations relying on the specific staple crop. For example, HarvestPlus (https://www.harvestplus.org) seeks to develop and distribute varieties of food crops (rice, wheat, maize, cassava, pearl millet, beans, and sweet potato) that have higher levels of iron, zinc, and provitamin A (one nutrient per crop) through an interdisciplinary global alliance of more than 400 scientific institutions and implementing agencies in developing and developed countries.

To be successful, three requirements must be met for implementation of biofortification. First, high and stable micronutrient density must be combined with high crop yield and productivity at similar costs to be attractive for farmers and consumers. Second, efficacy for human health must be demonstrated – the micronutrient status of human subjects must improve with regular consumption of biofortified varieties. Thus, adequate micronutrient levels must be retained during storage, processing, and cooking, and these nutrients must be sufficiently bioavailable. Third, the biofortified crops must be adopted by farmers and consumed by those suffering from micronutrient malnutrition, which requires incentives, education, and an appropriate delivery strategy.

Through the combined efforts of HarvestPlus and CIP and their collaborating partners, more than 300 varieties of conventionally bred biofortified crops have been approved by varietal release committees in 40 developing countries and are being tested for release in an additional 20 countries (https://www.harvestplus.org and https://www.cipotato.org). Efficacy trials have demonstrated improved zinc, iron, and provitamin A status in malnourished populations, improved cognitive function and work performance, as well as lower morbidity^24–26^. The ultimate vision is for all national agricultural research institutes to make high mineral and vitamin density core, non-negotiable breeding traits.

Biofortified crops offer a rural-based intervention that initially reaches these more remote populations that comprise a majority of the malnourished in many countries. This is then extended to urban populations as production surpluses are marketed. Initial investments in agricultural research at a central location can generate high recurrent benefits as locally adapted biofortified varieties become available in country after country across time at low recurrent costs.

Conventionally bred biofortified crops can provide an extra 20 to ≥100% of the Estimated Average Requirement (EAR; median daily intake value estimated to meet the requirement of half the healthy individuals in a life-stage and gender group) for specific nutrients^7,27,28^. The average addition to the EAR is ~25% for zinc crops, 35% for iron crops, and >85% for provitamin A crops^7,27,28^ (https://www.harvestplus.org/content/estimated-average-requirements-provided-biofortification; Table 1).

**Table 1:** Estimated proportion of the EAR supplied by conventionally bred biofortified crops.

Crop	Target increment	Estimated proportion of the estimated average requirement provided	
		Before biofortification (%)	After biofortification (%)
Beans	44 ppm iron	45	85
Cassava	15 ppm provitamin A	0	100
Maize	15 ppm provitamin A	0	55
	12 ppm zinc	50	75
Pearl millet	30 ppm iron	50	80
Sweet potato	70 ppm provitamin A	0	≥100
Rice	12 ppm zinc	40	70
Wheat	18 ppm zinc	25	45

The values assume certain levels of per capita consumption, retention of nutrients in processing, storage and cooking, and bioavailability. Further information is provided in refs. ^7,27,28^. This table is adapted from HarvestPlus website with permission (https://www.harvestplus.org/content/estimated-average-requirements-provided-biofortification). Source data are provided as a Source Data file.

## Increasing multiple minerals using metabolic engineering

When the natural variation in sexually compatible germplasm is insufficient to achieve satisfactory micronutrient levels in a specific crop by conventional breeding, biofortification via metabolic engineering can offer a solution. In rice, this is the case for multiple micronutrients, including iron, provitamin A, and folates.

Increasing Fe and Zn content in rice, which is consumed by ~3.5 billion people, has a remarkable potential to alleviate micronutrient deficiencies. To provide an additional 30% of the EAR in women and children, the iron concentration in polished grain needs to be increased by 11 ppm from the baseline of 2 ppm to reach 13 ppm, assuming of 10% bioavailability; while for zinc, an increase of 12 ppm from 16 ppm will provide an additional 30% of the EAR, assuming of 20% bioavailability^7^. Micronutrient levels in polished grains are most relevant, as polished grains represent the energy-rich white rice which is consumed by the populations suffering from micronutrient deficiencies. Only a limited variation for iron in polished rice is available in germplasm collections, which limits conventional breeding for this trait. However, there is sufficient natural variation for zinc to reach a concentration of 28 ppm. Several high zinc rice lines, created through conventional breeding, have been released in Bangladesh, and were recently released or are under development for several additional countries (www.harvestplus.org).

In the abovementioned case where conventional breeding is constrained by limited natural variation, genetic engineering can be used to augment micronutrient content in crop plants. Identification of most of the key genes involved in Fe and Zn uptake, translocation, and storage^29,30^ has facilitated the development of enriched Fe and Zn rice by transgenic approaches^31^. For the same event, a significant increase in both Fe (up to 15 ppm) and Zn (to 45 ppm) in polished grain was successfully achieved in a high-yielding variety, without increased uptake of unwanted heavy metals (cadmium, arsenic, and lead)^32,33^. The level of Zn in this transgenic variety is significantly higher than in the released conventionally bred Zn cultivars.

Combining Fe and Zn traits with other micronutrients and agronomic traits in multiple high-yielding varieties is obviously desirable. With respect to mineral micronutrient enhancement, it should be emphasized that success of biofortification fully depends on both the level and bioavailability of the different minerals in the soil. Where the latter are insufficiently present in the substrate, foliar, or soil fertilization can be applied^34^. These agronomic strategies are complimentary to metabolic engineering and breeding technologies in poor soils.

## Filling in a biosynthetic gap towards provitamin A in rice

An example of how genetic engineering enables introduction of a micronutrient that is essentially absent in a crop species is Golden Rice, which is enriched in provitamin A (β-carotene)^35^. Rice leaves, and indeed the photosynthetic tissues of all higher plants, produce and accumulate β-carotene. However, non-engineered polished white rice has no detectable provitamin A carotenoids, as the outer layers of the kernel that do contain low amounts of this vitamin are removed during the polishing process. No naturally occurring rice varieties have been reported that accumulate β-carotene in the grain and could be used for conventional breeding, thus genetic engineering provides the only viable approach.

The second-generation Golden Rice (GR2E)^36^ has been found to accumulate up to 37 ppm dry weight total carotenoids in the polished grains immediately after harvest, although the concentration is dependent on the genetic background and reduces during storage after harvest. To make this trait suitable for cultivation in Asia, the transgenic GR2E rice required breeding into farmer-preferred *indica* rice varieties, yielding lines containing between 3.5 and 10.9 ppm dry weight total carotenoids in polished grains^37^. Even with these lower concentrations of provitamin A, it is estimated that 100 g of uncooked GR2E can supply 30−40% of the recommended daily allowance (RDA) of vitamin A for children^25^, at least 75% of which remains after cooking^38^.

Golden Rice has provided a number of lessons for future programs to develop and introduce transgenic biofortified crops. The process of moving from proof of concept to a product that can be used by farmers and consumers is difficult, as illustrated by the sequential evaluation of various transgenic GR2 events since 2006. Arguably, a more effective selection earlier in the process would have accelerated the establishment of events qualified for breeding. In addition, the challenges of working with a transgenic regulated product in countries with new or changing regulatory processes cannot be underestimated. These factors, combined with the high public profile of Golden Rice as both a standard bearer for plant biotechnology and a target of activists, have made for an unnecessarily long and arduous journey. However, there is reason for optimism. The December 2019 decision by the Government of the Philippines to authorize the direct use of GR2E in food, feed, and for processing^39^ is greatly encouraging for its eventual release. Submission of an application for commercial propagation of Golden Rice in the Philippines is anticipated in 2020. In addition to the import approvals already obtained in Australia, Canada, New Zealand, and the US^40–42^, an application for cultivation and food use was submitted in Bangladesh in November 2017^43^, where it remains under review.

The slow progress of Golden Rice in Bangladesh illustrates a fundamental problem for genetically engineered biofortified crops that do not also provide an agronomic benefit. Especially in countries with new and evolving regulatory frameworks, the authorization of a genetically engineered crop requires committed support among all key decision-makers, often at some political risk. New products must not only meet environmental, animal, and human health and safety standards, but must present a compelling value proposition that can garner the support of not only just nutritionists and ministries of health and family welfare, but also ministries of agriculture who wield significant influence in the decision-making process. Also critically important is how the value proposition is framed, and by whom, recognizing that local voices are the most authentic and persuasive.

## Reaching the RDA in single food servings

Inadequate intake of B-vitamins is highly prevalent in regions where rice is the main staple food. The natural variation of folates in rice is far too low to reach the necessary increase of at least 100-fold to meet the RDA value for pregnant women with one or two servings of 100 g of white rice^44^. The first successful example of a genetic engineering approach improving rice for B-vitamins is the 100-fold increase of folates (vitamin B9) in polished rice grains, reaching 1723 µg/100 g fresh weight, which is close to the RDA and more than the EAR in a single serving^45^.

Genetic engineering of the B-vitamin metabolic pathways has been successful in different staple crops. Increased levels of bioavailable vitamin B6 could be achieved in cassava storage roots^46^, while folate levels could also be significantly increased in potato tubers^47^ as well as wheat and maize grains^48^. Efforts to engineer the vitamin B6 metabolic pathway in rice have revealed limitations in increasing B6 vitamin levels in polished white rice; however, the same strategy was capable of elevating B6 vitamin levels in rice leaves^49^. Therefore, further research is needed to understand the factors currently impeding the genetic engineering of the B6 and other B-vitamin metabolic pathways in rice endosperm.

## Reducing or stopping post-harvest vitamin degradation

A potentially more effective or complementary breeding strategy for high vitamin levels in foods produced from biofortified crops is the reduction or complete prevention of vitamin degradation after harvest. Ensuring post-harvest storage stability of vitamins is of paramount importance, especially considering the longer storage at elevated temperatures, which is a typical practice in populations suffering from vitamin deficiencies^50^. Provided that sufficient fundamental knowledge on vitamin stabilization is available, engineering these traits in food crops can yield both increased vitamin accumulation as well as elevated stability. The long-term stabilization of vitamin B9 obtained by introduction of genes encoding folate binding proteins or altering folate structure to enable increased (post-harvest) stability are successful and encouraging examples^50^. Moreover, the combination of this B9 stabilizing strategy with the aforementioned folate biofortification approach has enabled the hyper-accumulation of folate (up to 150-fold of wild-type rice levels) in transgenic polished rice seeds^50^. Similar approaches might also help to increase vitamin B1 and B6 stability in the polished rice grain.

Like folates, all other vitamins are prone to breakdown during storage, with varying sensitivity depending on the specific vitamin and food matrix. A study of β-carotene corn in Zambia showed that provitamin A levels had dropped by 70% after 6 months^51^; similar losses were reported for polished Golden Rice^38^. Nevertheless, amounts remaining after degradation can still provide an additional amount of vitamin A in the diet, ~40% of the EAR, and the rate of degradation asymptotically approaches zero after a certain time point.

Different approaches have been shown to successfully stabilize provitamin A in engineered staple crops. In potato, β-carotene levels can be enhanced and stabilized by introduction of the *Orange(Or)* gene^52^. *Or* induces the formation of chromoplasts, which naturally function as a metabolic sink for carotenoid accumulation. Overexpression of *Or* together with the two other genes for β-carotene biosynthesis in GR2 enhanced β-carotene levels in polished rice to 26 ppm dry weight, though stability of the provitamin A still needs to be investigated^53^. Alternatively, engineering towards decreased carotenoid oxidation, an important form of provitamin A degradation, increased provitamin A stability in polished Golden Rice during storage^54^. Suppressing provitamin A breakdown has also been implemented in wheat to increase β-carotene levels 31-fold^55^. In another approach, combining engineering towards higher accumulation of tocochromanols, fat-soluble E-vitamins bearing antioxidant properties with the approach used in GR2 has shown that vitamin E can enhance the stability of provitamin A in sorghum grain, more than doubling its metabolic half-life^56^.

## Simultaneously increasing multiple nutrients

Combining, or stacking, multiple nutrient traits into high-yielding varieties is important and preferable over single nutrient enhancement^57^, similar to the routine stacking of transgenes for insect resistance and herbicide tolerance in cotton and maize^58,59^. Combining several nutrition-related genes from multiple parents, including genetically engineered crops such as Golden Rice, into a single genotype through conventional backcrossing is possible, but very time-consuming and laborious. Also, care must be taken to avoid undesired alteration of other favorable plant characteristics in the backcrossing process and to prevent segregation of transgenic traits in subsequent generations. Therefore, combining multiple traits through conventional breeding remains a challenge, particularly given that simultaneous selection for multiple traits becomes increasingly difficult as the number of traits increases.

Today’s transgenic technologies substantially reduce the time frame in which a multi-nutrient product can be engineered and identified. For example, a single locus multi-nutrient trait improvement has been recently accomplished by simultaneously increasing iron, zinc, and β-carotene content in polished rice^60^. This illustrates the relative ease with which multiple micronutrients (iron, zinc, and provitamin A in this case) can be tackled by insertion of a single DNA fragment into the rice genome. This approach is encouraging and opens new perspectives for developing multi-nutrient staple crop varieties in one step. It exemplifies an effective and sustainable way of addressing multiple micronutrient deficiencies that often co-occur in affected populations^11,61^. Metabolic engineering allows design of strategies to jointly boost an array of different micronutrients (e.g. iron, zinc, vitamin A, and vitamin B9), whilst also taking the aforementioned stability into account. Moreover, with the advent of genome editing technologies, it is now possible to insert a gene cassette at a pre-defined location (safe harbor) in the genome of a crop plant, thereby avoiding adverse effects such as yield losses^62^. On a cautionary note, researchers need vigilance to recognize unintended metabolic consequences of biofortification. For example, provitamin A biofortification of cassava and sweet potato significantly reduces dry matter content of storage roots^63^. Since dry matter is a major factor influencing food texture, it is an essential component in product adoption. Enhancing other micronutrients might have a similar negative impact, possibly exacerbated in multi-biofortified crops.

As a health intervention, even single-nutrient biofortification is inherently cost-effective due to very low recurrent costs once varieties have been developed at a central location and widely distributed. Other micronutrient interventions, such as supplementation and industrial fortification of processed foods, involve much higher recurrent costs and are not directed at solving the underlying cause of micronutrient deficiencies, which is that agricultural systems are not producing affordable supplies of required minerals and vitamins^7^. Multi-nutrient biofortification can realize even more substantial economies of scale. While genetically engineered biofortified crops are associated with substantial development and regulatory costs, they are estimated to be highly cost-effective, often more than conventional or complementary approaches^64^. Ex-ante evaluation of multi-nutrient rice (provitamin A, folate, zinc, iron) in China, for example, has confirmed the potential, long-term cost-effectiveness^65^. Aside from synergies through aggregated health impacts and cost savings (from development to dissemination), multi-nutrient biofortification can lead to much higher market coverage, as competition between several single-nutrient biofortified varieties is avoided.

## Simultaneous improvement of nutritional and agronomic traits

**Fig. 1:**
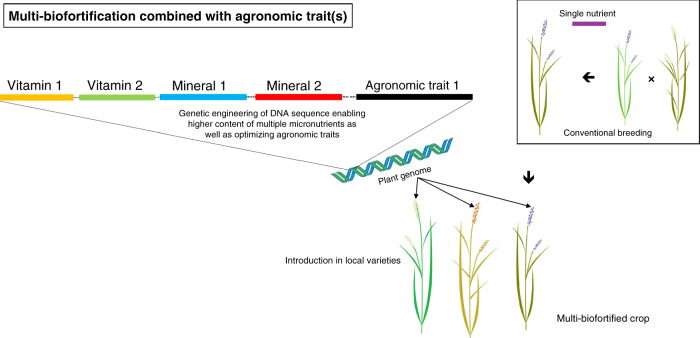
Multi-biofortification in combination with agronomic traits. Genetic engineering enables creation of a single DNA cassette, harboring information to allow increase in multiple micronutrients (vitamins and/or minerals) as well as favorable agronomic traits, possibly in combination with (conventional) breeding. The cassette can be targeted to a specific genomic location in multiple local cultivars of a crop plant of interest, e.g. rice.

A multi-nutrient trait locus stacked with genes of interest can be easily transferred to farmer-preferred crops, either by breeding or direct transformation in shorter time period. Genetic cassettes can now combine many more stacked genes and therefore it is conceivable that simultaneous micronutrient improvement of staple crops is no longer limited to three traits. Also, gene stacks for nutritional traits can be combined with important agronomic traits in the same genetic locus, such as pest and disease resistance (e.g., resistance to bacterial blight, army worms, brown plant hoppers, or stem borers in rice) or abiotic stress tolerance (e.g., drought, heat, salinity, cold, flooding tolerance; Fig. 1). This will make it more attractive for farmers to adopt nutritionally improved staple crop varieties and therefore accelerate the percentage of the total supply of rice (or other staple food crops) that is biofortified. Single insert gene cassettes using proven genes can be customized for specific regional needs or consumer preferences, which might also be favorable from a regulatory point of view. In an era of global climate change, the single locus-multiple trait approach combining nutritional traits with stress resilience genes can provide region-specific solutions for sustainable agriculture with benefits both for farmers and consumers. In doing so, genetically engineered biofortified crops might acquire a higher economic value that positively impacts their regulatory review^66^.

Small-scale farmers and low-income rural communities in Africa and Asia are the ones suffering most from micronutrient deficiencies and are likely also the ones that will be hit hardest by climate change and related weather extremes. Genetic engineering technologies alone are not a magic bullet that will solve these problems, but they can help considerably in making small-farm systems more productive, resilient, and nutrition-sensitive. In the past, with conventional breeding approaches, breeding for higher yields was sometimes associated with lower crop robustness and lower nutritional value. Such tradeoffs can now be avoided. Another important advantage of genetic engineering is the much greater speed of breeding, especially against the background of climatic changes leading to new weather and pest infestation conditions that are not always predictable. Studies suggest that harnessing new breeding technologies for combining nutritional and agronomic traits can lead to large economic and social benefits in developing countries and represent an important step towards sustainable food security^67,68^.

## Proposed actions and policy interventions

UN-SDG2 of ending all forms of hunger and malnutrition by 2030 needs much swifter actions than those that are currently deployed. Implementation of biofortified crops, either obtained by conventional breeding, genetic engineering, or their combination can substantially contribute to reach this goal. Fortunately, the speed at which novel crops can be developed is more rapid than ever due to advances in gene cloning and genome editing technologies^69,70^. Together, these technologies provide the means of minimizing trait loss due to instable genomic context and optimization of gene expression levels.

Reaching health-relevant nutritional targets is often not possible by conventional breeding. Presently, the combination of genetic engineering and conventional breeding is the most powerful approach when aiming at multi-nutrient crops. There are currently no examples of sufficient nutrient enhancement via genome editing approaches. Genome editing technologies alone are indeed not solving the problem until we have a better understanding of the tissue-specific activity of relevant gene promoters or unless mutations can be identified that boost the reaction rate of required enzymes. Genetic engineering should go hand in hand with conventional breeding and could be assisted by novel breeding technologies for site-specific (safe harbor) insertion.

Conventionally bred biofortified crops have been extensively tested for efficacy, even showing improved functional outcomes such as lower morbidity, improved cognitive performance, and work capacity. These trials have taken into account nutrient losses due to processing and cooking, and during storage. The presumption is that higher nutrient levels, as consumed, in transgenic foods will show even better nutrition and functional outcomes, although trials are needed to confirm this. Modern breeding technologies including genetic engineering should be applied to elite germplasm for combining nutritional and agronomic traits to produce high quality-high yield crops.

**Fig. 2:**
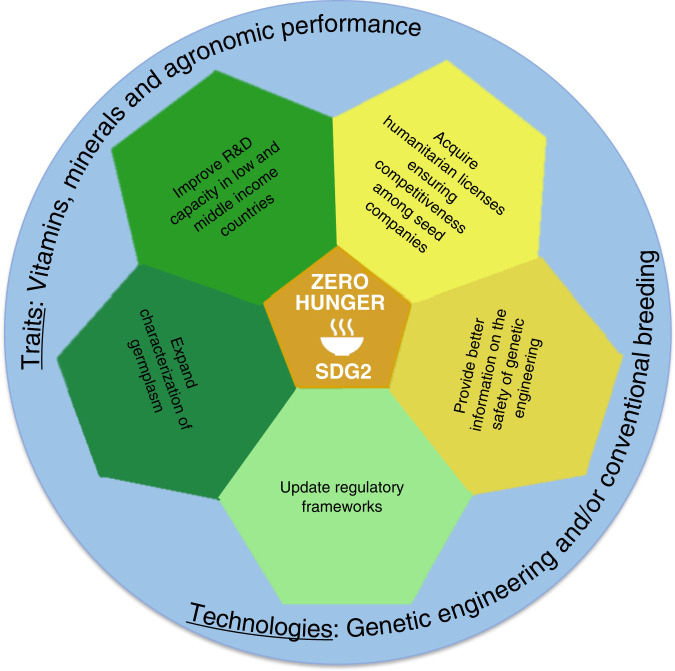
Proposed actions and policy interventions. Achieving zero hunger (UN-Sustainable Development Goal 2, UN-SDG2) requires actions (green-yellow) using different tools and trait/technology combinations (blue). Yellow-labeled actions specifically aim to achieve better accessibility by the poor, whilst green-labeled actions aim to acquiring more efficient product development (light green involves genetic engineering only, dark green includes both genetic engineering and conventional breeding).

In this context, therefore, the following specific actions and policy interventions are recommended (Fig. 2):

1. There is a need to expand germplasm collections as well as screen existing germplasm to better characterize natural diversity of micronutrients as the benefit could be two-fold: availability of genotypes for conventional breeding approaches and genetic material to better understand vitamin biosynthesis and micronutrient accumulation *in planta*.
2. Key to making biofortified crops successful is their accessibility in a way that vulnerable population groups and smallholder/subsistence farmers can affordably receive these biofortified crops. Release of the biofortified crop products needs to be preceded by ethically approved and well-designed dietary studies supporting their effect. Humanitarian licenses will be needed from institutions that own intellectual property rights on these crops as well as genome editing technologies and genetic elements used in biofortification approaches. This enforces independence from seed companies, which will allow for a better public perception of genetic engineering technology, especially if clear health (consumer-related) and economic (farmer-related) benefits are demonstrated.
3. Single locus, multi-nutrient trait strategies facilitate qualitative product improvement without altering plant health and creating environmental issues. The safety of genetically engineered crops for consumers and the environment was endorsed by Science Academies worldwide (http://nas-sites.org/ge-crops/category/report/) and by over 100 Nobel laureates^71^. However, there is still a need to provide independent and science-based information to the public on safety of genetically modified crops.
4. Regulatory frameworks for genetically engineered foods and crops need to be established, or updated and modernized in different countries, to acknowledge three decades of experience and evidence of their safety. Regulations should be proportionate to the level of risk, considering both familiarity and history of safe use. The May 2020 announcement by the U.S. Department of Agriculture’s Animal and Plant Health Inspection Service (APHIS) to exempt certain categories of gene edited plants from its regulatory oversight signals an important shift from process-based regulation to an approach that focuses on the new traits themselves. Many countries (e.g. in Africa) either do not have a regulatory framework or very restrictive for the release of transgenic crops. The establishment of local non-profit organizations (e.g. Cornell Alliance for Science) aiding the public, media and policymakers in understanding the implications of introduction of multi-biofortified crops, both from a health and a socio-economic perspective, should ease the process towards cultivation of biofortified crops by small-scale farmers and release of the products to low-income rural communities. Publicly funded bodies, preferably with additional financial backing of charitable organizations, coordinated by governments in concert with local academics could ensure a non-profit route towards population groups in need.
5. Capacity needs to be built in developing countries to increase impact by implementation of technologies in farmer- and consumer-preferred crop varieties. Empowering research and product development capacities in regions where metabolically engineered biofortified crops are needed is the key to facilitate technology adoption^72,73^. Regional research centers and universities, for instance, can focus on the development of genetic transformation and genome editing tools in local recalcitrant genotypes. Likewise, local agricultural institutions, particularly in regulatory-benign countries, can be involved in the development phase of the work under the form of field trials. This is particularly important for crops requiring extensive breeding programs due to a long life-cycle, asynchronous flowering, and high heterozygosity.

## Conclusions

Metabolic engineering, combined with conventional breeding, can catalyze a much more rapid approach to the realization of zero hunger and to reducing malnutrition by crop biofortification along the successful path paved by HarvestPlus and other public institutions. Based on decades of experience with the widespread and safe production of transgenic maize and soybean as food crops, the consensus among Academies of Science globally is that transgenic crops introduced thus far are not harmful to the environment and are safe for human consumption. Considering the fact that many crop species, including sweet potato, yams, and banana, are naturally transformed by T-DNA from Agrobacterium^74^, common sense should prevail when governments decide on legal frameworks for the release of transgenic crops. Such decisions should be solely driven by the determination to improve human welfare and sustainability. However, scientists have an important role to play beyond the walls of their labs, notably in transferring of knowledge and making sure that regulatory decisions are based on ratio. Academies of Science over the globe can, together with the publicly funded bodies advocated above, function as a motor in the process of knowledge transfer towards policymakers.

**Fig. 3:**
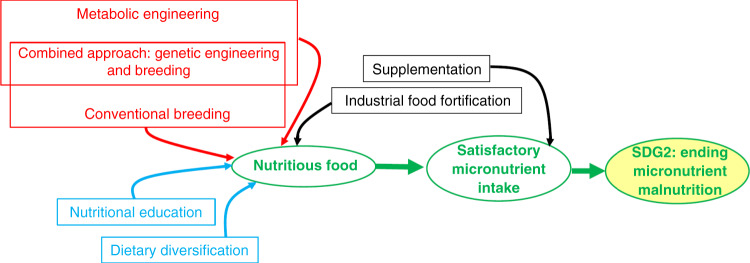
Ending malnutrition requires concerted efforts from different angles. Biofortification (red), dietary interventions (blue), and fortification or supplementation (black) can help affected populations to reach sufficient micronutrient intake, leading toward eradication of micronutrient malnutrition and its related diseases. Metabolic engineering of crops for better micronutrient content is an efficient, cost-effective strategy complementary to conventional breeding and offers several benefits. It allows faster trait introduction, including the stacking of multiple traits as a single genetic locus in a crop genome, and achieves increases in essential minerals and vitamins while simultaneously improving vitamin stability. Conventional breeding is far less constrained by regulatory issues however, which today unnecessarily slow the deployment of novel genetically engineered varieties with increased micronutrient content to improve human health. Lessons learned from Golden Rice have already led to a better understanding of multilevel stakeholders and the strong need for joint efforts of bringing an important nutritive trait from the lab to farmers and consumers^66^. If adopted multi-nutrient biofortified crops currently under development can help millions and particularly women and children sustainably, complementing other nutrition interventions. We emphasize that ending malnutrition needs increased efforts on a wide array of interventions, including making healthy diets more affordable through higher incomes and lower food prices. Together with nutrition education, these efforts result in greater dietary diversification. Increasing incomes of the poor and adding relatively expensive vegetables, fruits, pulses, and animal products to diets are important for improved micronutrient status and other better nutrition-related outcomes, but will only be realized over several decades. Supplementation and industrial food fortification are important interventions in the short and medium run, but biofortification can and should play a larger role as a cost-effective and agriculture-based improvement of human health. Achieving sustainable change needs a substantial increase of investments, demanding global governmental action in all parts of the chain, from research and development to education and dissemination. Public funding of such efforts is of paramount importance.

Biofortification, in combination with dietary diversification and nutrition education, holds great potential to eradicate micronutrient malnutrition and increase global human health (Fig. 3). The next gene revolution should focus on sustainable solutions for malnutrition, as part of a humanitarian intervention, concerted with educational efforts as a cornerstone to halt population growth, improve living standards, and bring about global peace.

## Data Availability

Source Data are provided with this paper.

## Source data

Source Data
